# Supplementary material for: Are carbon emissions trading and green financial instruments synergistic? -Comprehensive quantitative research based on content analysis
Source: PLoS One. 2024 Mar 7;19(3):e0298601. doi: 10.1371/journal.pone.0298601 (PMC10919601; doi:10.1371/journal.pone.0298601)
Supplement: S1 Data — (ZIP) [file pone.0298601.s001.zip › Data/地级市Z_F工作报告.zip!/║■▒▒/╗╞╕╘╩╨/└┤╫╘╣1⁄2╓┌║┼í╛┬φ┐╦╩2╛▌═°í┐_2006.docx]

各位代表：

　　现在，我代表市人民政府向大会作政府工作报告，请连同《黄冈市经济和社会发展第十一个五年规划纲要(草案)》一并审议，并请市政协各位委员和其他列席人员提出意见。

　　一、“十五”时期经济社会发展及2005年工作回顾 五年来，在市委的正确领导下，全市上下紧紧围绕“赶超全省发展水平”和“十五”计划目标，坚持“全党抓经济，重点抓工业，突出抓招商，着力抓环境”，抢抓机遇，加快发展，成功应对非典、禽流感疫情和多种自然灾害的挑战，有效抑制经济运行和社会发展中出现的不稳定不健康因素，经济建设、政治建设、文化建设、社会建设都取得了新成就。

　　──经济发展速度和运行质效逐步提高。2005年，全市生产总值达到348.6亿元，按可比价格计算，五年年均增长7.6%,人均GDP达到5176元,其中一、二、三产业分别增长6.2%、8.3%和9.1%。全市完成财政收入29.2亿元，按可比口径五年平均增长9.6%。其中地方一般预算收入在农业税全免的情况下，达到13.1亿元，年均增长11%。农业产业化深入推进，农业综合生产能力明显提高，粮食生产下滑的局面得到扭转，主要农产品规模效益和优质率不断上升，农民从事农业生产的积极性得到有效发挥，农村经济活力大为增强。工业园区蓬勃发展，产业结构不断优化，基本形成了食品饮料、机械电子、医药化工、纺织服装、建筑建材等优势产业，产值过亿元的骨干企业发展到30家。商贸、旅游、金融、保险、通信和房地产等第二产业得到长足发展。

　　──项目建设和招商引资取得重大突破。“十五”时期，全市完成固定资产投资583亿元，五年年均增长12.7%。尤其是城乡基础设施投资多、变化大。交通建设，完成一级公路80公里、二级公路620公里、通乡油路897公里、通村公路1678公里，鄂黄长江大桥、江北一级公路建成通车、沪汉蓉快速铁路、大广高速公路和武英高速公路相继开工;能源建设，保证了农村电网改造一、二期工程和城网改造工程的按时竣工，争取了白莲河抽水蓄能电站、大别山火电厂和黄冈天然气项目的动工兴建;水利建设，重点整险加固了长江干堤和9座大型水库、11座中型水库，改造扩建了3个大型灌区，加强了骨干泵站建设;通信建设，重点加强了网络建设，实施了“红色覆盖工程”和“农村电话“村村通工程”;城镇建设，完善配套了一批标志性工程和便民利民工程，增强了城市的综合功能。招商引资取得突破性进展。近三年，全市累计引进项目2974个，总投资额468亿元，实际到位资金143亿元一批国内外知名企业落户黄冈，使之成为我市“十五”时期经济发展最醒目的亮点。

　　──龙头经济与县域经济竞相发展。高昂市区龙头，突出发展工业经济和第三产业，市区主要经济指标增幅持续高于全市平均水平，市直规模以上工业增加值由全市第七位上升到第二位，市级财政收入由全市第四位跃居第一位。各县市认真贯彻“一主三化”方针，千方百计招商引资、激活民资、争取国资，集中力量建设工业园区，突出特色培育支柱产业和骨干企业，形成了县市区之间比学赶超的发展态势。

　　──改革开放不断深化。机构改革、行政审批制度改革和公共财政体制改革取得实效。国有资产管理体制改革和企业改革深度推进，市县乡村所属公有制企业已基本完成以民有民营为取向的产权制度改革。农村税费改革、乡镇综合配套改革、国有农场管理体制改革、粮食流通体制改革、投融资体制改革、事业单位改革取得新的进展。对外开放水平进一步提高。五年累计完成外贸出口5.94亿美元，年均增长8.6%;累计吸收外商投资2.34亿美元，年均增长19.2%。积极推进“四个一体化”，加快了融入武汉城市圈的进程。

　　──社会事业全面进步。加大政策支持和财政投入力度，促进了各项社会事业加快发展。全市预算内义务教育经费由2000年的2.7亿元增加到2005年的7.5亿元，年均增长29.5%;筹集资金6573.4万元，为43.6万名农村贫困学生免费提供教科书、免除杂费，为贫困家庭寄宿制学生提供生活补助;筹集资金1.4亿元，改造农村中小学危房48.1万平方米。高等教育、职业教育、成人教育不断壮大，普通高校本专科在校生达到3.4万人，其中高职1.9万人。积极开展科技特派员制度试点，实施省级以上产业化示范项目50多项，科技创新能力增强，科技对经济增长的贡献率达到50%。文化事业和文化产业加快发展，优秀文艺创作人才和优秀精神产品不断涌现。大力推进卫生改革和发展，重点推进新型农村合作医疗改革试点、公共卫生和医疗救治体系建设以及血防机构改革，全市疾病预防和医疗救治体不断健全，艾滋病、血吸虫病等重大传染病和地方病防治取得新成绩。高度重视人口、资源、环境工作，低生育水平得到巩固，出生人口性别比偏高的问题得到有效遏制，环境质量总体保持稳定，国土管理在保护资源和保障发展方面发挥了重要作用。民政、民族、宗教、对台、外事、侨务、人防、档案、方志、经研、保密、妇女、儿童、老龄、残疾人等各项工作都取得机关报成绩。

　　──为群众谋利益取得实效。千方百计扩大就业再就业，五年累计新增城镇就业岗位13.5万个，帮助困难群体再就业16万人。着力完善社会保障体系，确保国有企业下岗职工基本生活费、离退休人员基本养老金、失业人员失业保险金和城镇贫困居民最低生活保障金按时足额发放。加大社会救助、救灾和扶贫工作力度，全市农村特困户救助对象达12.2万人，城乡贫困群众大病医疗救助1345人，较大幅度提高了各类优抚对象的抚恤标准，农村贫困人口平均每年减少近5万人。城乡居民收入持续增加，农民人均纯收入由“九五”末2081元增加到2005年的2644元;城镇居民人均可支配收入由4600元增加到6358元;年均递增6.7%;人均储蓄由1652元增加到3998元,年均递增19.3%。切实维护群众合法权益，坚决纠正在农村征地、城镇居民房屋拆迁、企业重组改制、企业违法排污等方面损害群众利益的行为，组织开展了清理拖欠等方面损害群众利益的行为，组织开展了清理拖欠进城务工人员工资等多项劳动保障执法检查，加强了医疗、教育等领域“乱收费”治理。加强安全生产管理，重特大事故明显减少。市政府每年承诺并认真办好8到10件实事，解决了不少事关群众切身利益的实际问题。

　　──精神文明建设和民主法制建设进一步加强。坚持用邓小平理论和“三个代表”重要思想武装广大干部群众，认真组织开展保持共产党员先进性教育活动，政府机关人员的思想政治素质明显提高。精神文明创建活动扎实开展，基层民主建设深入推进。积极推行政府决策科学民主化，制定并实施了《关于全面推进依法行政的若干意见》，加强行政执法管理，强化行政执法监督，加快了建设法治政府的步伐。建立健全社会预警体系和应急机制，提高了处置突发事件的能力。深入开展普法教育，公民法制观念进一步增强。加强社会治安综合管理，依法打击各种犯罪，加强和改进信访工作，积极化解各类矛盾，推动了平安黄冈建设。积极开展国防教育和“双拥”活动，支持部队建设，认真做好军队转业干部、复员退伍军人接收安置、优抚和政策落实工作，军政、军民关系进一步密切。认真落实党风廉政建设责任制，建立述职述廉述学制度和行政首长廉政谈话制度，强化反腐倡廉措施，加大审计监察工作力度，廉政建设进一步加强。

　　刚刚过去的2005年，全市上下乘势而上，开拓进取，着力营造良好的体制、机制和发展环境，着力推进农村改革与发展，着力培育市场主体和有竞争力的产业，着力发展市区经济和县域经济，着力推进对外开放和融入武汉城市圈建设，着力维护人民群众切身利益，着力解决经济社会生活中的突出矛盾和问题，全市经济社会进一步呈现出良好的发展态势。全市生产总值比上年增长9.3%,全社会固定资产投资增长21.9%，社会消费品零售总额增长12.1%，外贸出口增长23.2%，地方一般预算收入增长14.9%，城镇新增就业3.6万人;城镇居民人均可支配收入和农民人均纯收入分别增长8.4%、6.4%，均高于年初确定的预期目标。居民消费价格上涨3%，城镇登记失业率4.2%，人口自然增长率2.7‰ ，均控制在调控目标之内。这些成绩的取得，既为“十五”划上了一个圆满的句号，又为“十一五”发展奠定了良好的基础。

　　各位代表，“十五”时期我市改革开放和现代化建设取得的成就，来之不易。这是在党中央、国务院、省委、省政府和市委的正确领导下，全市人民团结拼搏、共同努力的结果;是历任黄冈领导打下的良好基础和市人大、市政协监督支持的结果;是驻黄部队、武警官兵，各民主党派、工商联、无党派人士，各人民团体和社会各界积极参与的结果;是上级有关部门、外地黄冈籍人士、国内外各界朋友尤其是来黄冈投资的外地客商鼎力相助的结果。在此，我代表市人民政府，向全市各条战线的广大干部群众，向所有关心和支持我市经济社会发展的海内外人士，致以崇高的敬意和衷心的感谢!

　　在肯定成绩的同时，我们也清醒地看到，当前经济社会生活中还存在不少困难和问题:我市仍属于一个欠发达地区，经济体制、经济结构、经济增长方式等方面存在的深层次矛盾尚未从根本上解决，二、三产业发展相对滞后，尤其是工业经济产业缺块头、企业缺龙头、产品缺拳头的问题仍很突出;城市化水平偏低，中心城镇尤其市区经济实力不强，带动能力较弱;地方财力不足，居民收入偏低，社会事业发展方面的欠帐较多，人口、资源、环境与就业再就业的压力很大;政府自身改革和职能转变尚未到位，社会管理和公共服务职能较弱，一些部门管理方式落后，办事效率不高，有的政府工作人员依法行政观念不强，还存在官僚主义、形式主义和腐败行为。对这些问题，我们一定高度重视，进一步增强忧患意识和责任感，知难而进、坚持不解地采取有效措施加以解决。

　　二、“十一五”时期经济社会发展的总体要求和奋斗目标

　　“十一五”时期是全面建设小康社会、赶超全省发展水平的关键时期。根据党的十六届五中全会、省委八届九次全会和市委二届十次全会精神，市政府经过深入调查研究，认真听取各方意见，制定了《黄冈市经济和社会发展第十一个五年规划纲要(草案)》。《纲要(草案)》对“十一五”时期我省经济社会发展面临的环境和条件进行了全面深入的分析。从外部环境上看，未来五年，世界经济正处于新一轮恢复和增长期，我国经济发展正处在新的经济周期的上升期、国际资本向中国及沿海产业向中西部转移加快、国家“促进中部崛起”和我省加快建设武汉城市圈的战略全面推进，将为我市加快发展提供重大战略机遇。从内在条件上看，近年来黄冈区位优势日益凸显，发展环境明显改善，市场主体和特色经济不断发育，一批知名企业纷纷落户和重点建设项目相继动工，为加快发展打下了良好基础。尤其是市委、市政府提出的“四抓”发展思路已成共识，全市上下聚精会神搞建设、一心一意谋发展，奋力赶超全省发展水平、加速实现富民强市目标的氛围已经形成。这将为黄冈经济进入新一轮快速增长期提供强有力的支撑。因此，我们完全有条件在未来五年发展更快一些、更好一些，必须继续抢抓机遇，乘势而上。

　　基于上述分析，《纲要(草案)》提出了“十一五”时期我市经济社会发展的总体要求和主要目标。总体要求是：坚持以邓小平理论和“三个代表”重要思想为指导，以科学发展观统领经济社会发展全局，全面贯彻落实党的十六届五中全会精神，切实把黄冈经济社会发展转入科学发展轨道;坚持“全党抓经济，重点抓工业，突出抓招商，着力抓环境”的工作方略，实施生态立市、工业强市、开放活市、特色富市、科教兴市发展战略，加快调整优化经济结构和转变经济增长方式，促进全市经济社会又快又好地发展，奋力赶超全省发展水平;坚持用发展和改革的办法解决长期积累的矛盾和前进中的问题，协调推进全市经济建设、政治建设、文化建设、社会建设，加速构建和谐黄冈。经过五年努力，使全市综合经济实力跃上新台阶，社会事业发展实现新进步，城乡人民生活水平得到新改善，民主法制建设和精神文明建设取得新进展。按照上述总体要求，《纲要(草案)》提出了未来五年全市经济社会发展的主要目标。主要目标中既有经济增长的预期目标，也有能耗下降的约束性目标;既有经济建设的目标，也有政治建设、文化建设和社会建设的目标。这几个方面的目标，是一个相互联系、相互促进的有机整体。这里，我着重从五个方面作几点说明：

　　一是促进经济又快又好地发展。始终坚持把抓好发展作为第一要务，坚持以经济建设为中心，坚持加快发展、科学发展。“十一五”时期，在结构优化、效益提高、消耗降低的基础上，力争生产总值年均增长10%以上，提前实现人均生产总值比2000年翻一番;地方财政一般预算收入年均增长12%;全社会固定资产投资年均增长15%，五年投资总额达到1230亿元;社会消费品零售总额年均增长12%;外贸出口年均增长15%;吸收外商投资年均增长12%，五年吸纳外商投资4.6亿美元;单位生产总值能源消耗比“十五”末降低20%左右。

　　二是加速产业结构优化升级。坚持规模扩张与结构调整并重，不断提升黄冈经济发展的竞争力。以加快形成食品饮料、机械电子、医药化工、纺织服装、建筑建材和森工等支柱产业为重点，强力推进新型工业化;以建设特色优质农产品生产加工区为目标，加强农业综合生产能力建设和生态环境建设，积极推进农业结构战略性调整和产业化经营;以发展现代物流业和新兴服务业为重点，提升第三产业的比重与档次。到2010年，三次产业比重调整为24.8:34.9:40.3。

　　三是推动城乡区域协调发展。积极适应行政和财政管理体制改革的新形势，着力高昂市区龙头、壮大市级经济，保持市区发展持续高于全市平均水平，增强市区的综合功能和带动能力。按照建设社会主义新农村的要求，逐步建立完善工业反哺农业、城市支持农村的政策体系。以融入武汉城市圈为契机，健全黄冈市域协调发展机制，形成“一心(黄冈市区)两翼(麻城、武穴)三带(京九沿线、长江沿岸、大别山腹地)”的鄂东城镇群。加快城镇化进程，2010年全市城镇化水平达到40%。

　　四是实现体制机制创新和科技创新。不断深化改革开放，完善现代企业制度和产权制度，更大程度地发挥市场在资源配置中的基础性作用，形成有利于落实科学发展观的体制机制。大力推进科教兴市和人才强市战略，不断提高自主创新能力。到2010年，关系全市经济社会发展全局的重大体制改革取得突破性进展;科技进步对经济增长的贡献率达到55%以上;各类人才总量达到61万人。

　　五是加强和谐社会建设。坚持以人为本，更加注重经济社会协调发展，更加注重维护社会公平与正义，更加注重民主法制建设。到2010年，全市九年义务教育在普及的基础上进一步巩固和提高，15岁及以上人口平均受教育年限提高到9年以上;人均期望寿命达到76.4岁，农村新型合作医疗覆盖率达到80%;文化事业、文化产业和文化强市建设取得重大进展;人口自然增长率控制在年均6‰以内;主要污染物排放总量控制在2005年水平，森林覆盖率和城市建成区绿化覆盖率分别达到45%和40%;5年新增就业岗位15万个，城镇登记失业率控制在4.5%以内，城镇养老、失业、医疗保险覆盖率达到90%以上;全市城镇居民人均可支配收入年均增长8%，农村居民人均纯收入年均增长6%。

　　三、“十一五”时期经济社会发展的重点和主要措施

　　(一)强力推进新型工业化。改变黄冈工业化滞后的状况，是加快黄冈发展的根本出路和长期任务，必须锲而不舍，强力推进。为此，《纲要(草案)》着重从三个方面提出了目标和措施。一是做大做强支柱产业。立足黄冈特色，发挥比较优势，重点支持食品饮料、机械电子、医药化工、纺织服装、建筑建材和森工等产业加快发展。力争到2010年，六大支柱产业的年增加值占整个第二产业的比重达到80%以上。二是突破性发展高新技术产业。坚持自主研发与引进、消化、吸收相结合，改造提升现有优势传统产业，加速培育壮大新兴产业，形成具有黄冈特色的高新技术企业和高新技术产品群，到2010年，高新技术产业增加值达到34亿元，占工业增加值的比重达到20%以上。三是整体提升工业竞争力。重点推进各类开发区、工业园区建设，加强体制、机制和管理创新，促进企业向园区聚集、资本向园区流动、人才向园区集中、政策向园区倾斜，使各类开发区和工业园区成为优势产业的载体。进一步调整企业组织结构和产品结构，提高产业集中度和产品关联度，力争全市有3个行业产值过百亿元，有2家企业产值过50亿元，23家企业产值过10亿元，93家企业产值过亿元;大力实施精品名牌战略，继续积极引进国内外知名品牌，加速争创有自主知识产权和地方特色的本地品牌。

　　(二)积极推进社会主义新农村建设。坚持 “生产发展、生活宽裕、乡风文明、村容整洁、管理民主”的要求，求新重建，统筹规划，分步实施，整体推进，力争使全市农村经济实力有明显增强，农民科技文化和思想道德素质有明显提升，农村社会事业发展有明显进步，村容村貌有明显改观。基层组织战斗力有明显提高。一是加快现代农业建设。继续调整农业经济结构，转变农村增长方式，推进农业标准化生产，发展高产、优质、高效、生态、安全农业，提高农业综合生产能力和市场竞争力。大力推进农业产业化经营，培育优质产品品牌，加强龙头企业和产业板块基地建设，使更多的农户进入农业产业化体系。围绕优势产业和特色农产品，继续加快建设“五带十区”，重点实施500万头生猪和500万亩速生丰产林、500万亩板栗、500万亩优质稻、500万亩油菜的“五个五百万工程”。二是全面深化农村改革。抓好农村综合改革，基本完成乡镇机构、农村义务教育、县乡财政管理体制改革等任务。同时，加快农村金融、农产品流通、土地征用制度等方面的改革。三是着力推进文明新村建设。坚持因地制宜、统筹规划、分步实施、稳步推进，高质量地抓好3000个自然村文明示范点建设，带动25%以上行政村建设“文明新村”。着力加强饮水安全、农田水利、乡村道路、农村能源、城乡电网、邮电通讯、广播电视等设施建设，实现电力、公路、邮政、电话、广播电视“村村通”，60%的行政村普及自来水。四是发展农村公益事业。加大农村教育、卫生、文化等投入，努力使全市80%的村建立健全农村合作医疗体系、农村社会化服务体系，养老保险、五保户集中供养等社会救助体系。五是建立和完善促进农民增收的长效机制。主要是，扩大畜牧业、水产业、花卉苗木业等产业规模，充分挖掘农业内部增收潜力;大力发展农产品加工、运销和农村服务业等非农产业;大力发展县域经济和城镇特色产业，促进农村劳动力转移;大力实施农村劳动力转移培训阳光工程，培训农民工达到100万人次以上，培养农村实用型人才达到25万人以上。六是坚持不懈地抓好扶贫开发。大力实施整村推进、产业扶贫、扶贫搬努力培养有文化、懂技术、会经营的新型农民;坚持整村推进，力争五年内基本解决现有25万极端贫困人口的温饱问题，帮助50万低收入人口实现稳定脱贫。

　　(三)加快发展和提升服务业。重点是,突破性发展旅游业，形成红色旅游、绿色旅游、古色旅游互联互动、相得益彰的特色旅游业新格,争创优秀旅游城市。力争到2010年，全市旅游总收入达到64亿元，旅游业在全市经济中的比重达到8%。加快发展信息服务业，推进信息服务业向数字化、宽带化、综合化、智能化方向发展。改造提升传统服务业，运用现代服务技术和经营方式全面改造商贸、餐饮等行业，加快发展现代仓储、配送加工、多式联运、商品批发和信息服务“五位一体”的现代物流业，形成便捷高效、竞争有序的市场格局。积极培育成长型服务业。支持促进金融保险业、房地产业和各类中介服务业的发展。

　　(四)统筹城乡和区域协调发展。一是着力高昂市区龙头。坚持市区一体，加快市区建设，发展市区经济，把市区建成经济较发达、功能完备、环境优美、特色鲜明的滨江园林城和历史文化教育名城。加强新城区建设，完善功能区布局，推进中心城区向东扩张。到“十一五”末，市区建成区面积达到40平方公里，城市人口达到40万人。二是加快发展县域经济。落实政策，创新体制，扩权让利，减少审批事项，创造良好环境，加大财政、金融、能源、项目、用地等支持力度，大力支持县域经济发展，力争经过五年的努力，涌现一批各具特色的经济强县，每个县市区要着力培植1—2个优势产业，建成1—2个投入5亿元以上的工业项目，形成几个税收过千万元的骨干企业。三是积极融入武汉城市圈。依照资源整合、优势互补、利益共享、联合协作、经济一体的原则，加强与圈内各市的联合与协作，搞好产业、设施、市场、科教、信息、政策等对接，融入大武汉，促进大发展，把我市建成绿色农产品生产加工区、现代制造业聚集区、高新技术吸纳区、区域性物流连锁配送区、中高级实用人才培养区、都市居民近郊休闲区。

　　(五)发展和完善基础设施与基础产业。能源建设，重点建设七大工程，即加快大别山火电厂、白莲河抽水蓄能电站建设，抓紧黄冈火电厂前期工作，积极推进龙感湖风力发电、红安秸秆和沼气等生物能发电项目，加快建设鄂东500KV输变电环形主网，完善市域电网，以及黄冈天然气项目建设。交通建设，重点建设“六网、七站、两港、一桥、一管”，构筑贯通大别山、沟通大长江、衔接大铁路、连接大城市，水陆畅通、边际沟通、便捷高效的大交通格局，实现公路交通网络化、铁路运输体系化、港口作业机械化、物流管理智能化。农业基础设施，以江河无病堤、山上无险库、平原无渍涝为主攻方向，加强一批重点水利工程建设，全面增强挡、排、灌、引、蓄能力;以实施大型商品粮基地、农业综合开发和“沃土工程”等项目为重点，提高农业综合生产能力。城市基础设施，以布局合理、设施配套、环境优美为目标，广泛吸纳社会资金投入市政设施建设，优先发展城市公共交通，建设完善排水、供水、供气设施，提高污水、垃圾处理集约化程度，加强“洁、绿、亮、美”工程建设，营造良好的人居环境。

　　(六)着力推进改革开放。一是深化经济体制改革。坚持市场化、民营化取向，深化企业改革，支持非公有制经济参与公有制企业改革，进入金融服务、公用事业、基础设施等领域。加快现代市场体系建设，大力发展土地、技术和劳动力等要素市场。深化财政、投融资、流通体制改革。二是深化行政管理体制改革。加快转变政府职能，优化政府机构设置。完善政府的经济调节职能，强化政府社会管理和公共服务职能，提高政府应对公共危机和风险的能力，把工作着力点更多地放在促进社会事业发展与和谐社会建设上。继续抓好事业单位改革，建立新型事业单位管理体制和运行机制。规范行政规则与程序，建立健全行政决策科学化民主化的基本制度。改进对政府部门及其公务员的绩效考核工作，形成有利于改进作风、狠抓落实的公务员管理体制和机制。三是积极扩大对外贸易。培植出口主导产业和骨干企业，扩大出口规模，优化出口结构，加强对外经济技术合作，进一步提高对外开放水平。四是坚持不懈地抓好招商引资。充分利用“两个市场、两种资源”，创新招商思路，提高引资质量，力争“十一五”期间每年招商引资达到80亿元。五是锲而不舍地优化发展环境，努力打造黄冈“低成本、快回报、高诚信、优服务、零障碍”的环境优势。

　　(七)实施科教兴市战略和人才强市战略。进一步深化科技体制改革，整合科技资源，推进科技进步和自主创新，加大科技成果转化力度，提高科技进步对经济增长的贡献率，争创全国科技进步先进城市。优先发展教育事业。以普通教育创名牌、职业教育出专才、高等教育上台阶为重点，整合教育资源，优化学校布局，增创教育优势，形成民办教育与公办教育优势互补、公平竞争、和谐发展的格局。积极推进人才管理体制创新。建立调动人才积极性、发挥人才创造性的激励机制，加强各类人才尤其是企业家队伍建设，着力优化人才环境，使黄冈成为创业者的乐园和人才的聚集地。

　　(八)坚持经济社会发展与人口资源环境相协调。这是实现可持续发展的重要保证。随着工业化、城镇化的推进，资源和环境压力还会不断加大，必须高度重视人口、资源、环境工作，大力发展循环经济，大力推进资源节约型、环境友好型社会建设。一是控制人口增长和提高人口素质。进一步落实计划生育奖励扶助制度，加强对流动人口计划生育的管理和服务，稳定低生育水平，有效治理出生人口性别比偏高的问题。二是大力发展循环经济。积极推进资源节约与综合利用，重点抓好节能节水节地节材。实行清洁生产审核制，积极开发和推广资源替代和循环利用技术，依法淘汰不符合国家产业政策、资源浪费、污染严重、技术落后的工业设备和产品。实行最严格的耕地保护制度，特别是加强基本农田保护。整顿和规范矿产资源开发秩序，抓好矿产资源的合理开发和保护。三是加强生态环境保护。采取有力措施，降低污染物排放总量。严格执行排放许可证、限期治理和建设项目环境影响评价制度，对严重污染环境、破坏生态的企业坚决关停。

　　(九)大力推进和谐黄冈建设。构建和谐黄冈，既是推进全市经济社会发展的重要目标，也是经济社会发展的重要保障。《纲要(草案)》着重强调要突出解决好人民群众最关心的就业、社会保障、医疗、文化生活、收入、安全问题。一是千方百计扩大就业。认真落实税费减免、小额信贷、财政贴息等一系列鼓励就业再就业的优惠政策，充分发挥中小企业和个体私营企业等非公有制经济对社会劳动力的吸纳作用，大力发展劳动密集型产业和社区服务业、城郊型农业等就业容量大的产业，培植新的就业增长点。建设功能齐全、运转高效的劳动力市场，加快建设各类职业介绍机构，提高劳动力输出的信息化水平。二是加快完善社会保障体系。完善养老保险制度以及城镇职工医疗、工伤、失业、生育保险制度;完善被征地农民基本生活保障政策;认真解决进城务工人员、灵活就业人员的社会保障问题;建立覆盖城乡的社会救助体系，提高城镇低保水平，探索建立农村低保制度。完善农村“五保户”供养、特困户救助、安全事故救助、重大疾病医疗救助、应急救助等社会救助制度。三是努力改善医疗卫生服务。重点是解决好群众看病难、看病贵的问题。四是注重提高人民群众收入水平。着力提高低收入者的收入，逐步扩大中等收入者比重。建立职工收入与经济效益同步增长的机制。建立工资支付保障制度，解决拖欠农民工工资问题。保持居民消费价格基本稳定，认真解决低收入群众的住房、医疗和子女上学等困难问题。五是丰富人民群众精神文化生活。继续抓好文明创建活动，加大政府对文化事业的投入，加强广播电视、体育等各项社会事业建设。六是努力促进社会稳定与和谐。完善社会管理的领导体制和机制，加强社会主义民主政治建设，妥善处理人民内部矛盾，做好信访工作，完善社会利益协调和社会纠纷调处机制，提高应对和处理突发事件的能力。加强社会治安防控体系建设，依法打击各种刑事犯罪活动，保障人民群众安居乐业。加大安全生产监管和执法力度，防止重特大事故发生。七是积极支持部队和国防建设。关心和支持驻黄部队、武警部队建设，深入开展国防教育，健全国防动员体系，抓好人民防空和民兵预备役工作。深入开展“双拥”活动，巩固军政军民团结。积极做好部队转业干部、复员退伍军人和离退休干部的接收安置和优抚工作。八是认真做好民族、宗教、对台、外事、侨务等工作。全面贯彻党的民族政策，积极引导宗教与社会主义社会相适应。充分发挥外事、侨务等部门的纽带作用，扩大对外交往与合作。积极发展残疾人事业，提高残疾人康复、教育、就业水平，关心和支持妇女、儿童、老龄等事业。认真做好经研、文史、档案、保密、方志等工作。

　　四、扎实做好2006年的工作，努力实现“十一五”的良好开局

　　今年是实施“十一五”规划的开局之年。做好今年的各项工作，意义重大。今年全市经济社会发展的主要预期目标是：地区生产总值增长10%以上;全口径财政收入和地方一般预算收入增长12%以上;全社会固定资产投资增长20%左右;社会消费品零售总额增长12%以上;居民消费价格指数涨幅控制在3%左右;外贸出口增长15%;实际利用外资增长12%左右;万元生产总值综合能耗下降4%;城镇居民人均可支配收入增长8%，农民人均纯收入增长6%;全年新增就业岗位3.1万个，城镇登记失业率控制在4.5%以内;人口自然增长率控制在6‰以内。实现上述目标，关键是按照市委二届十次全会和全市经济工作会议的部署，切实抓好各项工作的落实，抓好经济社会发展中的重点、难点及薄弱环节。

　　第一，切实转变经济增长方式，保持经济平稳较快发展。突出大企业和好项目两个关键，对优势企业和成长性企业实施重点扶持，引导新上一批行业领先的生产线和技改项目，推进产业链条延伸，带动发展一批中小企业，形成特色鲜明、竞争力强的企业集群。全市重点支持100家外商企业、100家民营企业、100个重点建设项目，加大协调服务力度，帮助解决实际困难，促进企业上规模，上档次，上水平，确保10家骨干企业进入全省中小企业50强或民营企业50强。结合城市规划建设，完善现有工业园区的配套基础设施，进一步增强承载能力。创新园区投入机制和经营机制，探索市场化运作、企业化经营路子，支持企业办园，鼓励民资建园。注重围绕园区核心企业，在引进配套企业的同时引进产业链下游企业，建设工业循环经济示范园。今年市区加快稳健工业园区和黄冈汽车工业产业园建设，各县市区重点建设1至2个工业园，使全市20个重点工业园区真正成为工业发展的亮点和新的经济增长点。二是切实加快社会主义新农村建设。着力抓好各项扶农惠农政策的落实;加大农业基础设施尤其是农田水利基础设施的投入改造力度。稳定发展粮食生产，加强高产农田建设，提高粮棉油的优质率。深入推进农业产业化经营，加强农产品加工龙头企业建设，抓好基地配套建设。继续完善粮食流通体制，积极构建现代粮食物流体系，保护和调动种粮农民积极性。加快乡村道路、电网、通信、安全饮水、沼气等工程建设，着力改善农村的生产条件和人居环境。加快小城镇建设，大力发展农产品加工、运销和农村服务业等非农产业。搞好第二次全国农业普查，为新农村建设提供科学依据。三是进一步发展和壮大服务业。切实把旅游业作为支柱产业来培育，力争今年接待国内外游客达到420万人次，旅游总收入突破18亿元。依托现有商贸企业和流通版块，加快交通、信息等基础设施建设，着力培育2至3家专业化、规模化、网络化水平较高的大型骨干物流企业。

　　第二，继续抓紧项目建设和招商引资，为经济社会发展提供有力支撑。按照国家投资方向，加大衔接力度，对已进入审批、核准程序的项目，加快完成项目审批，为争取国家投资创造条件;对未进入程序但国家有投资意向并可争取投资的项目，加大争取力度，力争有更多的项目纳入国家投资计划。大力优化投资结构，加大对工业特别是对现代制造业的投入，集中力量抓好大项目，力争有更多项目纳入国家和全省规划并早日开工建设。不断加大招商引资力度。继续坚持全民发动，全员招商，全力以赴扩大招商总量。同时，针对招商引资工作中存在的质量不高、规模不大、效益不明显的问题，积极从“招商引资”向“招商选资”转变。适度提高招商门槛，在投资方向、投资规模、投资强度、产出回报和生态环境等方面科学设置条件，逐步提高引资项目质量和档次，着力引进一批大项目、好项目和真老板。坚持政府主导，企业主体，在充分发挥党委、政府主导作用的同时，加强与企业的沟通，把企业推向招商引资的第一线。实行以外引外，以商招商。强化环境创新的各项措施，在全社会营造鼓励创业的浓厚氛围，力争创业发展环境有进一步的改善。

　　第三，坚持改革开放促发展，努力在深化改革和扩大开放上迈出新步伐。继续大力推进“三个退出”，全面完成国有企业改革为混合所有制企业的任务。完善国有资产监管体系，建立国有资本经营预算制度。全面放开搞活中小企业和乡镇企业，切实抓好改制不彻底企业的规范改制工作。高度重视研究解决免征农业税后出现的新问题，防止农民负担的转移和反弹。狠刹部门乱收费歪风，严肃查处违反政策加重农民负担的行为。进一步深化乡镇综合配套改革，下决心精简乡镇机构和人员，加快事业单位整合，妥善安排分流人员，巩固农村税费改革成果。抓紧制定鼓励各种经济成份、社会团体和个人投资的相关配套文件，努力扩大社会投资，引导社会资金投向经济社会发展的薄弱环节和重点领域。全面推行比较规范的部门预算，将所有政府收支活动全部纳入预算管理范围。全面实行国库集中收付，完善政府采购制度，深化收支两条线改革，强化非税收入收支管理，全面推行财政与编制政务公开。深入推进教育、科技、文化、卫生等社会领域改革，为社会事业加快发展提供有力的体制机制保障。全方位扩大对内对外开放，充分利用好两个市场、两种资源。创新利用外资方式，扩大利用外资领域，力争有更多的外商投资项目落户黄冈。着力培植出口主导产业和骨干企业，扩大出口规模，优化出口结构，加强对外经济技术合作。

　　第四，进一步加快市区建设和发展，奋力提升城市经济实力和整体形象。着力调整优化市区产业结构，通过内培盘活存量，通过技改扩大增量，通过外引扩大总量。继续集中精力、集中财力抓好黄州高新区和工业园区建设，加大林纸一体化等重点项目前期工作力度，支持汇源、太子奶、东联盛、深港纺织等项目扩建改造，大力发展市区教育事业、旅游产业和城郊农业，多渠道筹措资金，扩大投入，提高质量

　　和效益。持续强化城市规划、建设和管理，积极推进城市公用事业改革，有效发挥城投公司筹资、引资功能，实现多元筹资、滚动发展。深化市区发展重要问题的咨询研究，充分运用调研成果，着眼于从体制上增强市区发展的动力和活力。同时，坚持抓市区带全市、抓本级带下级，加强对县城经济发展的指导和引导，全面落实中央和省的支持政策，支持各县市发挥比较优势，发展有特色、有竞争力的产品和产业集群，形成市县共兴共荣的良好格局。

　　第五，加大社会事业的政策支持和财政投入力度，促进各项社会事业全面进步。深入实施科教兴市和人才强市战略，大力推动创新型社会建设，在全社会弘扬创新精神，强化创新意识，完善创新机制，培育创新人才，扎实推进传统产业高新技术化、高新技术产业化，力争形成一批自主知识产权和自主品牌，努力增强黄冈经济的竞争力。在继续抓好义务教育、高等教育的同时，大力发展职业教育，进一步落实教育费附加用于职业教育的政策，多渠道增加职业教育的投入。大力实施“农村薄弱学校改造工程”，促进城乡教育协调发展，努力实现教育公平。进一步完善公共卫生和医疗服务体系，改善农村医疗卫生条件，大力发展农村新型合作医疗和社区卫生服务;加强重大疾病预防控制，进一步加强医德医风建设和规范医疗服务价格，继续加快发展文化、广播电视、体育等社会事业。严格实行计划生育基本国策，落实计划生育奖励扶助政策，稳定低生育水平，加强对流动人口的计划生育管理和服务，加强对出生人口性别比失衡问题的综合治理。加强资源环境工作，努力建设资源节约型、环境友好型社会。

　　第六，切实解决群众生产生活中的实际问题，努力为人民办实事。重点办好八件实事：(1)加快农村公路建设，建成农村公路2000公里。(2)实施农村劳动力转移培训工程，当年培训27万人，转移20万人。(3)继续开展城乡贫困群众重大疾病医疗救助工作，落实救助资金，使城乡所有符合条件的对象得到救助。(4)实施50户以上自然村广播电视村村通工程，在全市1347个50户以上未通广播电视的自然村，建立稳定运行的广播电视公共服务体系。(5)实施“万村千乡”市场建设工程，使全市所有乡(镇)、村建有“农家店”，形成以城区店为龙头、乡镇店为骨干、村级店为基础的的农村消费经营网络。(6)加强艾滋病、血吸虫病、肺结核病防治工作，扩大新型农村合作医疗试点。(7)为市直持有《再就业优惠证》的大龄特困人员提供养老保险补贴，基本解决市直“零就业”家庭中有就业愿望和就业能力者就业。(8)建设黄冈市第二实验小学。(9)加大对职业教育的政策和财政投入，整合现有职教资源，使全市特别是市区职业教育的规模和质量取得突破性进展。(10)启动遗爱湖风景区建设,实施市区背街小巷改造工程，加快市区建设和发展。

　　第七，切实加强政府自身改革和建设。自觉接受人民代表大会及其常委会的法律监督，接受人民政协的民主监督，认真听取各民主党派、工商联、无党派人士和人民团体的意见，不断改进政府工作。加快转变政府职能，大力推进政企分开、政资分开、政事分开、政府与市场中介组织分开，强化政府的社会管理和公共服务职能，把财力等公共资源更多地投向社会管理和公共服务，把政府工作的着力点更多地放在推进社会发展与和谐社会建设上，真正还权于市场，服务于企业，让利于百姓。进一步完善公众参与、专家论证和政府决策相结合的决策机制。大力推进政务公开，发展基层民主，保证人民群众依法享有知情权、参与权和监督权。全面推进依法行政，加强和改进行政执法，着力解决行政执法职责交叉、多头执法等问题;规范行政执法行为，健全行政执法依据、程序公开制度，严格行政执法责任制度和过错追究制度。加强政风建设，以贯彻施行《中华人民共和国公务员法》为契机，进一步加强对公务员的教育、管理和监督，着力建设高素质的公务员队伍，使广大公务员特别是领导干部忠于职守，勤勉尽责，更好地服务基层，服务群众，服务市场主体。大力弘扬求真务实精神，树立正确的政绩观，重实际，说实话，办实事，求实效。进一步完善目标管理和重大事项督办制度，促进各项工作落实。加强廉政建设和反腐败斗争，健全教育、制度、监督并重的惩治和预防腐败体系。建立健全经济责任审计制度，对县处级以上领导干部全面实行经济责任审计，加大大案要案查处力度。

　　各位代表!黄冈的改革开放和现代化建设已经迈上了新的征程。做好“十一五”期间的各项工作，意义十分重大。让我们紧密团结在以胡锦涛同志为总书记的党中央周围，高举邓小平理论和“三个代表”重要思想伟大旗帜，在市委的领导下，坚持以科学发展观统领经济社会发展全局，解放思想，同心同德，求真务实，勇于创新，为开创黄冈改革开放和经济社会发展的新局面而努力奋斗!

　　序公开制度，严格行政执法责任制度和过错追究制度。加强政风建设，以贯彻施行《中华人民共和国公务员法》为契机，进一步加强对公务员的教育、管理和监督，着力建设高素质的公务员队伍，使广大公务员特别是领导干部忠于职守，勤勉尽责，更好地服务基层，服务群众，服务市场主体。大力弘扬求真务实精神，树立正确的政绩观，重实际，说实话，办实事，求实效。进一步完善目标管理和重大事项督办制度，促进各项工作落实。加强廉政建设和反腐败斗争，健全教育、制度、监督并重的惩治和预防腐败体系。建立健全经济责任审计制度，对县处级以上领导干部全面实行经济责任审计，加大大案要案查处力度。

　　各位代表!黄冈的改革开放和现代化建设已经迈上了新的征程。做好“十一五”期间的各项工作，意义十分重大。让我们紧密团结在以胡锦涛同志为总书记的党中央周围，高举邓小平理论和“三个代表”重要思想伟大旗帜，在市委的领导下，坚持以科学发展观统领经济社会发展全局，解放思想，同心同德，求真务实，勇于创新，为开创黄冈改革开放和经济社会发展的新局面而努力奋斗!
